# Patterns of biologic agent utilization among patients with rheumatoid arthritis: a retrospective cohort study

**DOI:** 10.1186/1471-2474-12-204

**Published:** 2011-09-19

**Authors:** Sarika Ogale, Elena Hitraya, Henry J Henk

**Affiliations:** 1 US Medical Affairs, Genentech, South San Francisco, CA, USA; 2 Health Economics and Outcomes Research, OptumInsight, Eden Prairie, MN, USA

## Abstract

**Background:**

The role of biologic therapies in the treatment of rheumatoid arthritis has expanded, but dosing patterns in the first versus subsequent lines of therapy have not been thoroughly explored.

**Methods:**

In order to describe patterns of biologic agent utilization among patients with rheumatoid arthritis, health care claims data on use of abatacept, rituximab, or the anti-tumor necrosis factor (TNF) agents etanercept, adalimumab, and infliximab in first- or subsequent-line settings were used to form patient cohorts. Variables included: starting dose (first administration or fill), maintenance dose (third administration or fill), average dose, dose escalation, inter-infusion interval, and discontinuation (gap in therapy > 60 days or switch). Time to discontinuation was assessed with Kaplan-Meier curves and Cox proportional hazards models.

**Results:**

Over 1 year, average (SD) doses of first-line etanercept (N = 1593; 45.4 [8.8] mg/week), adalimumab (N = 1040; 40.7 [10.4] mg/2 weeks), and abatacept (N = 360; 715.4 [214.5] mg/4 weeks) were similar to the starting and maintenance doses; the average infliximab dose (N = 538; 441.0 [209.2] mg/8 weeks) was greater than the starting and maintenance doses. Trends in the subsequent-line anti-TNF cohorts were similar. The percentages with a dose escalation or discontinuation were greater in the subsequent-line anti-TNF cohorts. The proportion with a dose escalation was greatest for the infliximab cohorts (61.2% first-line and 80.2% subsequent-line). The average period between abatacept infusions was 4.8 [1.4] weeks (4-week approved schedule); and 6.8 [2.6] months between rituximab courses (currently approved schedule is 6 months). Time to discontinuation was significantly shorter for subsequent-line than first-line anti-TNF therapy (median 9.7 vs. 12.5 mo; p < 0.001). The hazard ratio for discontinuing subsequent-line versus first-line anti-TNF therapy was 1.177 (p < 0.001).

**Conclusions:**

Subsequent-line anti-TNF therapy cohorts had higher rates of discontinuation, dose escalation, and shorter time to discontinuation than first-line anti-TNF cohorts.

## Background

Rheumatoid arthritis (RA) is a systemic autoimmune disease characterized by joint inflammation and progressive damage, as well as cardiac, pulmonary, ocular, and neurological complications [1,2]. These disease features are associated with increased disability and mortality [1,3]. The primary goal of RA treatment is remission or low disease activity [4,5], and therapies targeted to minimizing disease activity lead to achievement of other treatment goals, including pain control and prevention of joint damage and loss of function [4-8]. Current treatment strategies include long-term use of traditional and biologic disease-modifying anti-rheumatic drugs (DMARDs), as well as non-steroidal anti-inflammatory drugs (NSAIDs) and glucocorticoids.

Use of biologic DMARDs has increased over the past 15 years [9-11] and current recommendations advocate early use of biologic agents following an insufficient response to initial non-biologic DMARD therapy [4,12,13]. Several biologic DMARDs are available, and they differ in mechanism of action, route of administration, and frequency of administration. As of early 2009 (the end of the period investigated in this study), approved biologic DMARDs included the tumor necrosis factor (TNF) inhibitors etanercept, adalimumab, and infliximab, as well as a CD-20+ B-cell directed therapy, rituximab, and a selective T-cell costimulation modulator, abatacept. Etanercept and adalimumab are both self-administered as subcutaneous injections, whereas infliximab, rituximab, and abatacept are administered by intravenous infusion. Dosing intervals range from every week (etanercept) to a 2-dose course every 6 months (rituximab). These differentiating characteristics, as well as patient preferences together with clinical considerations are likely to affect the suitability of any of these medications for individual patients and thus therapy utilization patterns in real-world settings.

Studies have described patterns of biologic therapy utilization in RA, including characteristics of patients who receive biologics and what agents they receive [10,14], but dosing patterns in the first versus subsequent lines of therapy have not been thoroughly explored. With a goal of further understanding the use of biologic therapies in RA treatment, objectives of this study were to describe patterns of biologic utilization among commercially insured patients with RA, to compare utilization patterns of anti-TNF agents used as a first versus as a subsequent biologic agent, and to compare real-world dosing patterns with label recommendations.

## Methods

### Patient sample and data source

Commercially insured patients with evidence of RA were retrospectively identified in a large healthcare claims database affiliated with Innovus (now OptumInsight). The database includes enrollment information and medical and pharmacy claims from geographically diverse commercial health plan enrollees in the United States. To be eligible, patients were required to have at least 1 claim for a biologic agent of interest during the identification period 01 Feb 2006 through 31 Jan 2008. These agents were: adalimumab, etanercept, abatacept, infliximab, and rituximab. These medications represent the biologic agents approved for treatment of RA in the US during the study period, excluding the less frequently used biologic agent, anakinra. Biologic agents approved after the end of our study period (i.e., tocilizumab, cetrolizumab, and golimumab) were not included in this study. The self-injectable drugs adalimumab and etanercept were identified based on pharmacy claims, and the infused medications abatacept, infliximab, and rituximab were identified using medical (i.e., facility and physician) claims. Specifically, Healthcare Common Procedure Coding System (HCPCS) codes C9230, J0129, and J3590 were used to identify abatacept use, J1745 and S9359 were used to identify infliximab use, and J9310 was used to identify rituximab use. The date of the first use for any of these medications during the identification period was defined as the index date. If a patient had evidence of use of more than one of the medications of interest during the identification period, that patient could have multiple index dates and be included in more than one cohort, as described in the "Patient cohorts" section.

Patients were required to be continuously enrolled for 6 months prior to the index date (baseline period) and at least 12 months following the index date. The follow-up period extended from the index date until discontinuation or 1 year, whichever occurred earlier. At least one medical claim during the baseline period with RA diagnosis (ICD-9-CM 714.xx) in any position was required, and patients must have been aged at least 18 years as of the index date.

Patients were excluded if there was evidence of more than one biologic agent on the index date, evidence of receipt of the index medication prior to the index date, or diagnosis, at any time during the study period, of another indication for which the study biologics could be prescribed (Additional File 1, Table S1). Patients with > 50% more infusions or prescription fills per year than currently recommended in label guidelines for RA were excluded. Patients with pharmacy claims for adalimumab or etanercept indicating zero days supply or unusually high average daily dose (e.g., more than 40 mg/day for adalimumab or more than 30 mg/day for etanercept); or patients who had medical claims during follow-up for these normally self-administered medications, were excluded. Patients with medical claims for infliximab, abatacept, or rituximab that were missing units, and patients with pharmacy claims for these infused drugs during follow-up were excluded.

No identifiable protected health information was extracted or accessed during the course of the study. Pursuant to the Health Insurance Portability and Accountability Act [15], the use of de-identified data does not require institutional review board approval or waiver of authorization.

### Patient cohorts

Patients could be included in more than one cohort depending on their use of the biologic agents of interest. Cohort assignment was based on a hierarchical identification of the index medication. First, patients with medical claims for rituximab during the identification period were identified and the first administration date defined as the index date for rituximab. Second, patients with medical claims for abatacept were identified and the first use date defined as the index date for abatacept. Patients could be included in both the rituximab and abatacept cohorts. Third, patients with claims for anti-TNF agents (etanercept, adalimumab, infliximab) were identified. Patients whose first anti-TNF use occurred after rituximab or abatacept use were excluded from the anti-TNF cohorts.

Patients with an index anti-TNF were classified into first-line or subsequent-line anti-TNF cohorts based on evidence of anti-TNF use in the pre-index period. Patients with an index anti-TNF agent and no evidence of biologic use for RA prior to the index date (not restricted to the baseline period) were assigned to the first-line anti-TNF cohort. Patients with an index anti-TNF agent and a different anti-TNF agent in the pre-index period or who were previously in a different anti-TNF first-line cohort comprised the subsequent-line anti-TNF cohorts. The terms "first-line" and "subsequent-line" in this report refer specifically to anti-TNF use and do not reflect the use of other therapy for RA prior to initiating treatment with a biologic agent. Because rituximab and abatacept are most frequently used by patients who had an inadequate response to at least one anti-TNF, the rituximab and abatacept cohorts were not separated by "line" of biologic therapy.

### Study measures

The 6-month period prior to the index date was used to assess patient characteristics including comorbidities and prior use of therapy for RA. The burden of comorbidities was quantified using the Charlson comorbidity index [16]. Variables assessed in the post-index follow-up period included dose, dose escalation, discontinuation, and switching. These various descriptors were used to characterize actual doses used and changes over the course of treatment.

#### Biologic agent doses

Dosing was measured differently for infused and injected therapies. For infused therapies, the dose is recorded on the medical claim as the number of units billed for; these units were converted to milligrams. For injected therapies, an average daily dose was calculated based on the strength, quantity dispensed, and days' supply for specified fills (strength×quantity dispensed/days supply).

#### Biologic agent doses: starting dose

Starting and maintenance doses were determined for the anti-TNF and abatacept cohorts. "Starting" dose was defined as the recorded dose of the first administration for the infused anti-TNF products infliximab or abatacept, and as the average daily dose for the first fill of self-injected etanercept and adalimumab.

#### Biologic agent doses: maintenance dose

"Maintenance" dose was defined as the recorded dose of the third administration (infliximab and abatacept) or average daily dose for the third prescription fill (etanercept and adalimumab) among patients who had at least 3 infusions/fills before discontinuation.

#### Biologic agent doses: average dose

For anti-TNF cohorts with index medications dosed at regular intervals (etanercept, adalimumab), the average daily dose was calculated from the index date through follow-up (discontinuation or 1 year) among all patients in the cohort. For abatacept and infliximab cohorts, which have an initial loading period, an average daily dose was calculated based on all administrations from the third dose onward (post-loading period) among patients with at least 3 infusions during the follow-up period. The number of milligrams infused over the post-loading period was divided by the number of days from the third dose through the remainder of follow-up and this average daily dose was converted to dose per the recommended inter-infusion interval.

#### Biologic agent doses: last dose

The "last" dose, or the dose preceding discontinuation, switch, or at the end of the follow-up period was captured. The last dose was defined as the recorded dose of the last administration (infliximab and abatacept) or average daily dose for the last prescription fill (etanercept and adalimumab).

#### Anti-TNF agent dose escalation

Dose escalation was defined as a reduced interval between anti-TNF infusions or an increased average daily dose of an anti-TNF agent [17]. Patients using injected anti-TNF agents had a dose escalation if the average daily dose calculated for 2 or more consecutive prescription fills was greater than the maintenance dose. To avoid labeling the initial loading dose as an escalation, dose escalation for the infliximab cohort was defined as 2 reported doses greater than the maintenance dose, or 2 infusions within 7 weeks on 2 or more occasions. Escalation was assessed for patients in the anti-TNF cohorts who had at least 5 fills or infusions.

#### Infusion intervals

For patients in all infused therapy cohorts (infliximab, rituximab, abatacept), dosing was described based on the number of infusions and the time between infusions. The number of infusions was tabulated from follow-up period medical claims with evidence of index agent administration. The number of days between infusions was determined in order to compare timing with label guidelines [18-20]. For infliximab and abatacept cohorts, the loading period (time to complete the first three infusions) and infusion intervals during the post-loading period (from the third infusion onward) were assessed separately. The time between infusions was calculated from among patients with more than one infusion.

Rituximab is administered in 2-dose courses and the number of days between doses and between courses was calculated. A course was defined as receipt of a second infusion within 30 days. The number of days between courses was determined among patients with more than one course, and was based on the time between the first infusion of the first course and the first infusion of the second course.

#### Discontinuation of the index biologic agent

The proportion of patients discontinuing therapy and the time to discontinuation were determined for the anti-TNF and abatacept cohorts. "Discontinuation" was defined as a prolonged gap in supply of the index medication or when a patient had evidence of starting a non-index biologic medication during the follow-up period. The allowed gap in supply was initially defined as up to 60 days, and a sensitivity analysis allowing a gap of up to 180 days was also conducted. Patients with supply gaps longer than these allowed periods or who had evidence of non-index biologic agent use during the follow-up year were considered to have discontinued index therapy. For etanercept and adalimumab, the discontinuation date was defined as the last prescription fill date plus the days supply of that fill. For the infused therapies infliximab and abatacept, the discontinuation date was defined as the last infusion date plus the recommended interval before the next administration, specifically, 8 weeks for infliximab and 4 weeks for abatacept [18,19]. If a patient had evidence of starting a non-index biologic medication during the follow-up period, the discontinuation date of the index therapy was the date of the first fill or infusion of the new medication. The rituximab cohort was not included in the analyses of discontinuation because a retreatment interval was not specified on the product label during the study period. The rituximab label currently recommends a 6 month interval between courses.

#### Switching of biologic agents

The proportion of patients in each cohort who switched biologic therapy, i.e., had evidence of non-index biologic therapy use during the follow-up year was determined. The assessment period for switching covered the year following the index date, regardless of gaps in supply of the index therapy.

### Statistical analysis

Patient characteristics were analyzed descriptively by cohort. Means and standard deviations are provided for continuous variables and numbers and percentages are provided for dichotomous and polychotomous variables. Kaplan-Meier survival plots were used to describe the time-to-discontinuation and a Cox proportional hazards model was constructed to compare time-to-discontinuation between anti-TNF agents used in the first- versus subsequent-line settings while controlling for demographic (age, sex, region, insurance type) and clinical characteristics (Charlson comorbidity index, baseline methotrexate, NSAID, or corticosteroid use). Time-to-discontinuation was calculated based on the index date and discontinuation date without limiting the follow-up to 1 year. We used p < 0.05 to define statistical significance in the analysis of time to discontinuation. The Kaplan-Meier curves and Cox proportional hazard model were fit by STATA 10.0 (StataCorp, College Station, Texas) and all other analyses were conducted using SAS 9.1 (SAS, Cary, North Carolina).

## Results

### Cohort characteristics

Demographic and clinical characteristics of the patient cohorts are shown in Table 1. Across all cohorts, 59.3% to 67.7% of patients were aged 45 to 64 years. The source database population is comprised heavily of patients in the South region, and, as shown in Table 1, the geographic distribution of the database is reflected among patients with RA who were included in the study. Comorbidity scores were similar across the anti-TNF cohorts and were slightly higher among patients receiving abatacept or rituximab, suggesting greater comorbidity burden. In the baseline period, more than half of patients in every cohort used corticosteroids or methotrexate, and NSAID use was also common (Table 1). Median follow-up times ranged from 555 days (rituximab) to 661 days (subsequent-line adalimumab), indicating that at least half of patients in every cohort had at least 18 months of follow-up.

**Table 1:** Baseline patient characteristics

	First anti-TNF			Subsequent anti-TNF				
			
	Etanercept (N = 1593)	Adalimumab (N = 1040)	Infliximab (N = 584)	Etanercept (N = 258)	Adalimumab (N = 353)	Infliximab (N = 156)	Abatacept (N = 418)	Rituximab (N = 239)
Age; mean (SD)	49.9 (12.4)	50.1 (12.0)	52.8 (11.4)	50.0 (10.9)	49.9 (11.2)	49.1 (11.6)	52.8 (11.8)	52.7 (11.3)
Male; n (%)	346 (21.7)	245 (23.6)	145 (24.8)	56 (21.7)	64 (18.1)	31 (19.9)	69 (16.5)	48 (20.1)
Quan-Charlson score; mean (SD)	1.3 (0.8)	1.3 (0.9)	1.4 (0.9)	1.4 (0.8)	1.4 (0.9)	1.3 (0.8)	1.5 (1.0)	1.7 (1.1)
Medications; n (%)								
Oral and injectable corticosteroids	946 (59.4)	665 (63.9)	413 (70.7)	189 (73.3)	249 (70.5)	109 (69.9)	313 (74.9)	177 (74.1)
Methotrexate	833 (52.3)	606 (58.3)	401 (68.7)	142 (55.0)	177 (50.1)	89 (57.1)	213 (51.0)	120 (50.2)
NSAIDs	739 (46.4)	522 (50.2)	291 (49.8)	126 (48.8)	188 (53.3)	75 (48.1)	209 (50.0)	104 (43.5)
Antimalarials	274 (17.2)	192 (18.5)	104 (17.8)	41 (15.9)	65 (18.4)	23 (14.7)	41 (9.8)	26 (10.9)
Other DMARDs^a^	263 (16.5)	169 (16.3)	93 (15.9)	49 (19.0)	57 (16.1)	21 (13.5)	78 (18.7)	42 (17.6)
Immunosuppressants	22 (1.4)	14 (1.3)	3 (0.5)	2 (0.8)	7 (2.0)	6 (3.8)	22 (5.3)	7 (2.9)
Gold salt	1 (0.1)	1 (0.1)	0 (0)	1 (0.4)	1 (0.3)	1 (0.6)	2 (0.5)	1 (0.4)
Biologics; n (%)								
Etanercept	0 (0)	0 (0)	0 (0)	0 (0)	276 (78.2)	81 (51.9)	71 (17.0)	38 (15.9)
Adalimumab	0 (0)	0 (0)	0 (0)	166 (64.3)	0 (0)	62 (39.7)	60 (14.4)	42 (17.6)
Infliximab	0 (0)	0 (0)	0 (0)	57 (22.1)	55 (15.6)	0 (0)	141 (33.7)	54 (22.6)
Abatacept	0 (0)	0 (0)	0 (0)	0 (0)	0 (0)	1^b ^(0.6)	0 (0)	31 (13.0)
Rituximab	0 (0)	0 (0)	0 (0)	0 (0)	0 (0)	0 (0)	11 (2.6)	0 (0)
Follow-up (days); mean (SD)	656.9 (219.1)	664.1 (215.8)	685.4 (210.2)	677.9 (191.1)	691.1 (207.9)	675.2 (216.0)	636.4 (190.2)	617.0 (178.9)

^a^cyclophosphamide, d-penicillamine, leflunomide, sulfasalazine.

^b^J3590 (unclassified biologics) was included among the codes used to identify abatacept use; one patient had a claim with J3590 prior to the abatacept approval date and it was assumed that the code was used to refer to biologic use unrelated to rheumatoid arthritis.

DMARD, disease-modifying antirheumatic drug; EPO, exclusive provider organization; HMO, health maintenance organization; NSAID, non-steroidal anti-inflammatory drug; POS, point-of-service; PPO, preferred provider organization.

### Biologic agent doses

The doses received by patients with anti-TNF and abatacept biologic therapy are described in Table 2. For the self-injected therapies etanercept and adalimumab, the mean starting dose and maintenance dose were similar to the approved label dose for both first-line and subsequent-line cohorts. Appropriate infliximab and abatacept dosing is dependent on patient weight, which was not available in the dataset, but the mean starting and maintenance doses are presented in Table 2 for comparison. The average doses calculated for etanercept, adalimumab, and abatacept for the specified intervals are similar to the starting and maintenance doses, whereas the average infliximab dose per 8 weeks (the recommended infusion interval), was greater than the starting or maintenance doses, especially among patients who received infliximab after failure of a previous anti-TNF agent (Table 2).

**Table 2:** Dosing of anti-TNFs as first or subsequent line biologic therapy and abatacept

		First anti-TNF			Subsequent anti-TNF			
			
		Etanercept (N = 1593)	Adalimumab (N = 1040)	Infliximab (N = 584)	Etanercept (N = 258)	Adalimumab (N = 353)	Infliximab (N = 156)	Abatacept (N = 398)
*Label dose *[18,19,40,41]		*50 mg/week*	*40 mg/2 weeks*	*3 mg/kg^a^/infusion*	*50 mg/week*	*40 mg/2 weeks*	*3 mg/kg^a^/infusion*	*500-1000 mg^b^/infusion*

Starting dose^c^	mean ± SD	48.8 ± 18.3 mg/week	43.0 ± 19.7 mg/2 weeks	313.9 ± 98.7 mg/infusion	49.4 ± 17.6 mg/week	43.2 ± 13.8 mg/2 weeks	311.8 ± 101.7 mg/infusion	756.8 ± 350.1 mg/infusion

Maintenance dose^d^	Eligible (n)	1229	799	528	191	248	143	347
	mean ± SD	48.9 ± 13.1 mg/week	42.1 ± 10.4 mg/2 weeks	328.6 ± 109.0 mg/infusion	48.0 ± 6.6 mg/week	45.0 ± 13.7 mg/2 weeks	340.6 ± 107.6 mg/infusion	732.7 ± 221.0 mg/infusion

Average dose^e^	Eligible (n)	1593	1040	538^f^	258	353	144^f^	360^f^
	mean ± SD	45.4 ± 8.8 mg/week	40.7 ± 10.4 mg/2 weeks	441.0 ± 209.2 mg/8 weeks	47.8 ± 19.0 mg/week	45.4 ± 14.9 mg/2 weeks	556.2 ± 694.1 mg/8 weeks	715.4 ± 214.5 mg/4 weeks

Dose escalation	Eligible^g ^(n)	112	139	279	25	48	97	--
	Percentage of patients	11.1%	21.7%	61.2%	15.9%	24.1%	80.2%	--

^a^Patient weight.

^b^Recommended doses are 500 mg for patients < 60 kg, 750 mg for patients 60 to 100 kg, 1000 mg for patients > 100 kg.

^c^Starting doses for etanercept and adalimumab defined as the average daily dose of the first fill, which was converted to milligrams per the label treatment interval by multiplying by the appropriate number of days. The starting infliximab and abatacept infusion doses are reported as milligrams administered.

^d^Maintenance dose defined as the recorded dose of the third administration (infliximab and abatacept) or average daily dose (converted to milligrams per the recommended treatment interval) for the third prescription fill (etanercept and adalimumab) among patients who had at least 3 infusions/fills before discontinuation (> 90 day gap).

^e^Average daily dose calculated over the 1-year follow-up period (etanercept, adalimumab) or for the post-loading period (infliximab, abatacept) and converted to the label treatment interval by multiplying by the appropriate number of days.

^f^Requires at least 3 infusions.

^g^Requires at least 5 prescriptions fills or infusions.

^h^Last dose defined as the recorded dose of the last administration (infliximab and abatacept) or average daily dose (converted to milligrams per the recommended treatment interval) for the last prescription fill (etanercept and adalimumab).

### Anti-TNF agent dose escalation

The proportion of patients with a dose escalation was greater for the infliximab cohorts (61.2% for first-line and 80.2% for subsequent use) than for the other anti-TNF groups (Table 2). For all therapies, the percentage with dose escalation was greater for the subsequent-line biologic cohorts than the first-line cohorts, with that difference most pronounced for patients receiving infliximab (Table 2).

### Infusion intervals

Among patients who had infused biologic therapy, the number and timing of administrations provides additional information about treatment patterns. Over the follow-up period, a mean (± SD) of 7 ± 3 infusions was observed per patient among those in either the first- or subsequent-line infliximab cohorts, 3 ± 1 infusions were captured for the rituximab cohort and 10 ± 4 infusions were noted for the abatacept cohort. According to their prescribing information, the first 3 doses of infliximab and abatacept should be administered within a shorter time frame than later maintenance doses. For infliximab, these loading doses are scheduled for 2 and 6 weeks after the initial infusion. For patients with infliximab as a first-line biologic and at least 3 infusions (n = 538), 42.2% completed the 3 loading doses within 6 weeks after the initial infusion and a total of 78.1% completed the 3-dose series within 8 weeks. The proportion of patients who received the loading doses in the recommended period was higher for patients receiving infliximab as a subsequent-line biologic; among those with at least 3 infusions (n = 144), 47.2% completed the loading doses within 6 weeks after the initial infusion and a total of 88.2% had all 3 doses within 8 weeks.

**Figure 1:**
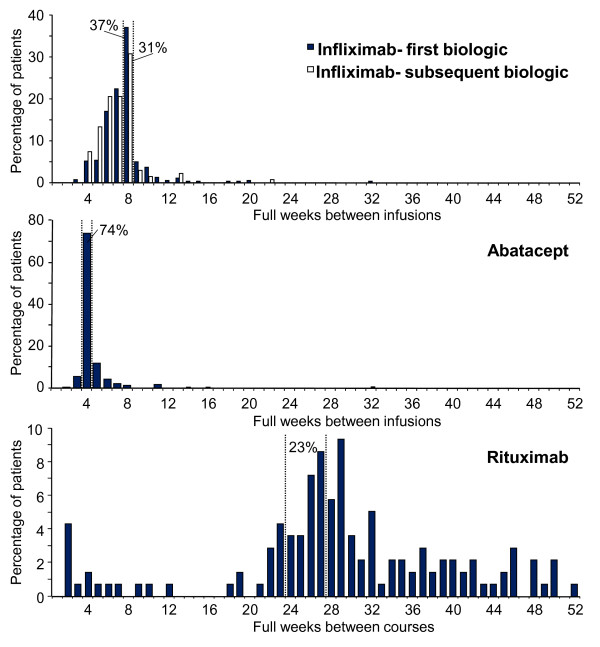
**Time between infusions**. The percentages of patients with the indicated mean infusion intervals during the post-loading period are shown among those with at least 4 infusions in the infliximab cohorts (first line infliximab n = 506; subsequent line infliximab n = 136) and the abatacept cohort (n = 344), and the percentage of patients with the indicated mean time between infusion courses among those with at least 2 courses is shown for the rituximab cohort (n = 139). Dashed lines indicate the current product label intervals (the course interval for rituximab was added to the label after the study period).

Following the loading period, infliximab is indicated for infusion every 8 weeks as per the label. The average time between infusions in the post-loading period in our study is shown in Table 3. The mean dosing interval was shorter for patients with infliximab as a subsequent-line biologic than for those who used it first-line and in both settings the average interval was shorter than the label recommendation. As shown in Figure 1, the most frequently observed average dosing interval for both the first- and subsequent-line infliximab cohorts aligned with the recommended 8 weeks (37.2% and 30.9%, respectively), although 50.4% (first line) and 61.8% (subsequent line) of individuals had a shorter than recommended average period between infusions.

**Table 3:** Recommended and observed time between infusions for intravenous biologics

	First-line infliximab^a^ (n = 506)	Subsequent-line infliximab^a^ (n = 136)	Abatacept^a^ (n = 344)	Rituximab^b^ (n = 139)
Label interval	8 weeks	8 weeks	4 weeks	6 months
Observed interval; mean (SD)	7.82 (2.19) weeks	7.39 (2.09) weeks	4.75 (1.42) weeks	6.8 (2.6) months

^a^Interval between doses starting with dose 3, among patients with at least 4 infusions in the follow-up period.

^b^Interval defined as the number of days between dose 1 of first course and dose 1 of subsequent course.

The abatacept loading period should be completed within 4 weeks of the initial infusion. For patients in the abatacept cohort who had at least 3 infusions (n = 360), 39.2% completed these loading doses within 4 weeks. Another 31.9% completed the 3-dose loading series between 4 and 6 weeks after the initial infusion. In the post-loading period, the average abatacept dosing interval was longer than the label indication (Table 3), but most patients' (73.8%) mean infusion interval aligned with the recommended 4-week duration (Figure 1).

Rituximab is administered in 2-dose courses, with the doses separated by 2 weeks and courses separated by 6 months. Among patients who had at least 2 rituximab infusions administered as a course (n = 200), the mean number of days between the infusions was 15.7 ± 3.0. Figure 1 shows that, among patients who had at least 2 courses (n = 139), 23% had an average between-course interval of 6 to 7 months (24 to 27 full weeks), 21% had an average interval of less than 6 months, and 56% had an average interval of more than 7 months.

### Discontinuation

When "discontinuation" was defined as a gap of more than 60 days or starting a non-index biologic agent, the proportion of patients that discontinued each subsequent-line anti-TNF therapy was greater than the proportion that discontinued the respective first-line anti-TNF treatment during the 1-year follow-up period (Table 4). The proportion of patients discontinuing anti-TNF treatment was highest for adalimumab in both settings. The proportion of patients receiving abatacept who discontinued was within the range of subsequent-line anti-TNFs. When the gap used to define discontinuation was extended to more than 180 days, the overall proportion discontinuing decreased, but the relative pattern remained the same (Table 4). The proportion discontinuing from subsequent-line anti-TNF therapy was still higher than from first-line therapy, and the adalimumab cohorts still had the highest proportion discontinuing among the anti-TNF cohorts (42.6% for first-line and 56.4% for subsequent-line).

**Table 4:** Percentage of patients discontinuing anti-TNF first- or subsequent-line biologic therapy and abatacept

	First anti-TNF			Subsequent anti-TNF			
		
Discontinuation definition	Etanercept (N = 1593)	Adalimumab (N = 1040)	Infliximab (N = 584)	Etanercept (N = 258)	Adalimumab (N = 353)	Infliximab (N = 156)	Abatacept (N = 398)
60 day gap or non-index biologic start	49.9%	52.9%	39.6%	52.7%	63.7%	45.5%	53.1%
180 day gap or non-index biologic start	36.7%	42.6%	33.4%	43.8%	56.4%	41.7%	18.0%

**Figure 2:**
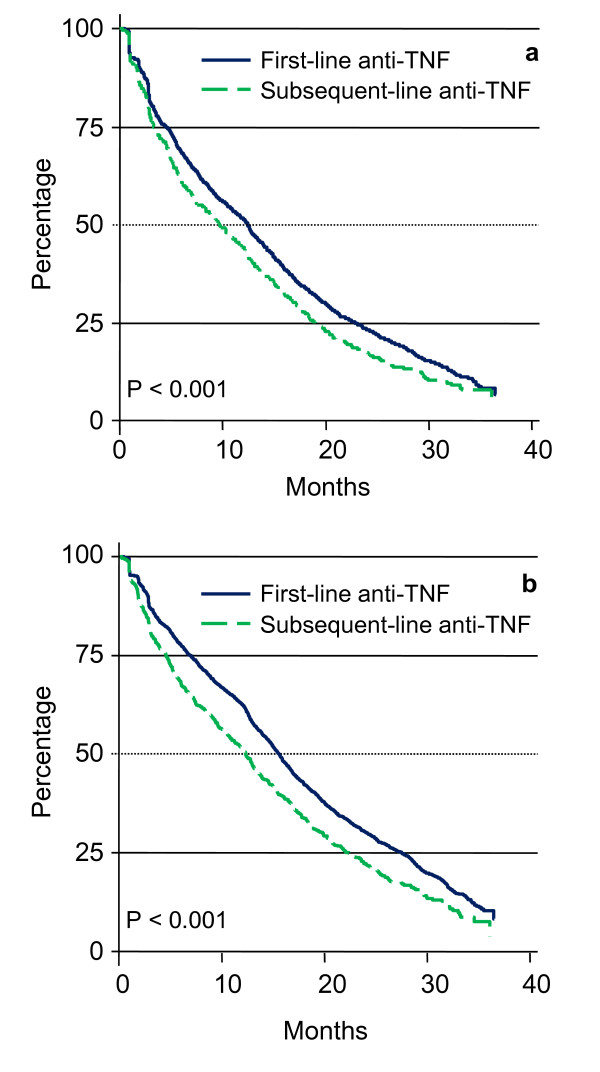
**Time to discontinuation of first and subsequent anti-TNF therapy**. When discontinuation was defined as: (**a**) a gap in therapy of more than 60 days or starting a non-index biologic agent, the median time to discontinuation was 12.5 months for first-line therapy and 9.7 months for subsequent-line therapy (p < 0.001); or (**b**) a gap in therapy of more than 180 days or starting a non-index biologic agent, the median time to discontinuation was 15.6 months for first-line therapy and 12.3 months for subsequent-line therapy (p < 0.001).

The Kaplan-Meier curves depicting time to discontinuation for first and subsequent-line anti-TNF therapy are shown in Figure 2. When assessed with a 60 day gap allowance (Figure 2a), time to discontinuation was significantly shorter for subsequent-line anti-TNF therapy than first-line anti-TNF therapy (p < 0.001). The median time to discontinuation of first and subsequent-line anti-TNF therapy, respectively, was 15.3 and 13.1 months for infliximab (p = 0.164), 12.3 and 9.7 months for etanercept (p = 0.103), and 10.9 months and 7.3 months for adalimumab (p < 0.001). The median time to discontinuation for abatacept was 10.8 months. The percentage of patients who remained on first-line anti-TNF therapy at 1 year was 51.4%, and 23.3% had continued with therapy at 2 years (note that continuous enrollment was required for 12 months). For the cohort of patients who used a subsequent anti-TNF therapy, 44.1% remained on therapy at 1 year and 17.4% remained on therapy at 2 years. When the allowed gap for discontinuation was defined as 180 days, median time to discontinuation increased by 0.4 to 4.9 months across all cohorts (Figure 2b).

Cox proportional hazards models for discontinuation (more than 60 day gap or start of non-index biologic agent) of anti-TNF agents are shown in Table 5. Compared with first-line anti-TNF therapy, risk of discontinuation was greater for subsequent-line therapy while controlling for demographic and clinical characteristics (combined anti-TNF cohorts hazard ratio [HR] 1.177; p < 0.001). Among specific anti-TNF therapies, however, only subsequent-line adalimumab was associated with a significantly higher risk of discontinuation (HR 1.238; p = 0.002). Risk of discontinuation remained significantly greater for subsequent-line anti-TNF therapy when a 180-day gap was allowed (HR 1.280; p < 0.001), but with this definition risk of discontinuation was significantly greater for subsequent-line etanercept (HR 1.246; p = 0.005) as well as for subsequent-line adalimumab (HR 1.300; p < 0.001). Risk of subsequent-line infliximab discontinuation was not significantly elevated versus first-line using either the 60-day (HR 1.140; p = 0.214) or 180-day (HR 1.201; p = 0.086) gap allowance.

**Table 5:** Cox proportional hazards model of time-to-discontinuation (more than 60 day gap in days supply or start of non-index biologic agent) of anti-TNF therapy

	Overall (N = 3984)	p value	Etanercept (N = 1851)	p value	Adalimumab (N = 1393)	p value	Infliximab (N = 740)	p value
Subsequent anti-TNF (reference first anti-TNF)	1.177	< 0.001	1.121	0.133	1.238	0.002	1.140	0.214
Age (years)	0.990	< 0.001	0.989	< 0.001	0.987	< 0.001	1.004	0.325
Female (reference male)	0.884	0.006	0.827	0.005	0.870	0.063	1.066	0.529
Region (reference Northeast)								
Midwest	0.954	0.545	0.874	0.218	1.005	0.973	1.198	0.386
South	1.016	0.829	0.886	0.222	1.066	0.618	1.366	0.113
West	0.930	0.370	0.791	0.043	1.095	0.520	1.068	0.758
Plan type (reference HMO)								
PPO	0.985	0.821	1.039	0.697	0.875	0.232	1.128	0.493
POS	0.873	0.288	0.668	0.050	1.152	0.516	0.864	0.578
EPO	1.028	0.621	1.025	0.767	0.943	0.520	1.321	0.045
Indemnity	0.902	0.136	0.847	0.109	0.806	0.060	1.344	0.083
Baseline treatment								
Methotrexate	0.836	< 0.001	0.871	0.013	0.832	0.003	0.801	0.014
NSAIDs	0.897	0.003	0.858	0.006	0.922	0.184	0.890	0.180
Corticosteroids	1.050	0.205	1.054	0.354	1.083	0.218	1.000	0.997
Baseline Charlson score	1.112	< 0.001	1.105	0.006	1.105	0.006	1.123	0.031

NSAID, non-steroidal anti-inflammatory drug; HMO, health maintenance organization; PPO, preferred provider organization; EPO, exclusive provider organization.

### Switching

Similar to discontinuation, the proportion of patients with evidence of non-index therapy use during the follow-up year, i.e., patients who switched medication, was greater among subsequent-line anti-TNF users than those in the first-line anti-TNF cohorts. For first-line anti-TNF agents the percentage of patients who switched to another therapy ranged from 12.0% (etanercept) to 15.4% (adalimumab). For subsequent-line anti-TNFs, the percentages ranged from 22.4% (infliximab) to 38.2% (adalimumab). For the non-anti-TNF therapies, 23.4% switched from abatacept and 17.5% switched from rituximab at some point during the follow-up year.

## Discussion

Our descriptive analyses suggest that, on average, the starting doses for biologic therapies for RA are in accordance with the label recommendations. However, a substantial proportion of patients who received infliximab had evidence of a dose escalation during follow-up. The higher rates of dose escalation, discontinuation, switching, shorter dosing intervals, and shorter time to discontinuation among patients previously exposed to anti-TNF therapy are suggestive of reduced effectiveness or poorer tolerance of subsequent anti-TNF agents after failure of the initial anti-TNF therapy.

The observed treatment patterns are consistent with results of previous studies showing reduced response with subsequent anti-TNF use compared with the first anti-TNF therapy as measured by changes in disease activity or treatment continuation [21-24]. Finckh et al. [25] have suggested that aspects of therapy utilization including dose escalation and discontinuation may be indicative of acquired resistance to therapy. Some previous studies have indicated that the response to subsequent anti-TNF therapy after failure of a first might still be acceptable for some patients [23,24,26], but the presence of alternative therapies compels further comparisons of these treatments within the context of real-world sequential administration practices. It has been suggested that introducing therapy with a non-anti-TNF mechanism of action may provide greater benefit than a subsequent anti-TNF medication [27]. In clinical trials, rituximab [28], abatacept [29,30] and tocilizumab [31] in combination with methotrexate were shown to be superior to methotrexate alone in patients who had an inadequate response to an anti-TNF agent. However, these studies did not compare the response achieved with these alternative agents against that achieved with a subsequent anti-TNF agent.

Finckh et al. compared a therapy providing an alternative mechanism of action, rituximab, with subsequent anti-TNF therapy following an inadequate response to a first anti-TNF and found a better clinical response with rituximab [32,33]. They further examined the effectiveness of these subsequent-line therapies in subgroups of patients with differing reasons for switching from the first anti-TNF agent, and found that the improvement in disease activity was significantly better among patients treated with rituximab versus a subsequent anti-TNF agent when the reason for switching was inadequate response [32]. When patients switched due to other reasons (including adverse events), the improvement in disease activity was similar between patients who received rituximab and those who received a subsequent anti-TNF agent [32]. In contrast, Blom et al. found no association between reason for discontinuation of the first anti-TNF agent and response to subsequent anti-TNF therapy [34].

A few studies have provided data regarding treatment patterns with biologic RA therapies. Using the same definition of dose escalation as we applied here, Gilbert et al. investigated dose increases among patients first treated with infliximab or etanercept [17]. Similar to our findings, they reported that approximately 58% of first-line infliximab users and 18% of patients receiving etanercept had a dose increase within 1 year [17]. Using different operational definitions, Nair et al. [35] found that 45% of commercially-insured patients with evidence of infliximab as a first-line biologic therapy had a dose escalation (difference between first and final doses) in the first year of therapy and, in a report based on a Dutch registry of patients with RA who started a first anti-TNF agent, Blom et al. [36] found dose increases in 36% of infliximab patients, 12% of adalimumab patients, and 8% of etanercept patients. Although our estimates for the comparable cohorts are based on a different definition of escalation, the overall pattern is the same; i.e., a high proportion of dose increases was observed among patients on infliximab and a relatively low proportion among those on etanercept. Although infliximab use was associated with a high proportion of dose escalations, we also observed that it had the longest continuation, suggesting that patients on infliximab tended to increase their dose rather than switch to another medication.

Studies of persistence or continuation with biologic therapy have produced varying results. Tang et al. [37] reported that, among US patients using anti-TNF biologic therapy in combination with methotrexate, those on infliximab had the longest duration of medication use and those on adalimumab used it for the least amount of time. Similarly, we found that infliximab use was associated with the longest median time to discontinuation and lowest discontinuation rate and that adalimumab was associated with the highest discontinuation rate and shortest time to discontinuation among first-line anti-TNF therapies. In contrast, data from a Danish registry showed that risk of withdrawal among patients using a first-line anti-TNF was greater for those on infliximab than for those using another anti-TNF therapy [38] and Italian registry data indicated that discontinuation rates were also relatively higher for patients who used infliximab [39]. Our patients showed a higher rate of discontinuation from subsequent-line anti-TNF therapy than those in a UK national registry [23]; in that study, 73% of patients who switched to a second anti-TNF remained on the new therapy through a mean of 15 months of follow-up. These differences indicate that a range of outcomes are possible for RA patients receiving anti-TNF therapy and the variability could reflect differences in preferences and practices across different countries. Comparisons between our study and these registry studies are also limited by the lack of clinical data in our database (e.g., disease severity).

Our study provides an illustration of biologic therapy utilization among patients with RA, but, like all claims-based analyses, the results must be interpreted in the context of several limitations. Claims are collected for payment purposes, not research; thus they are limited in the degree to which they represent a patient's true medical history. For example, claims-based evidence of a prescription fill does not confirm that a patient used the medication or used it appropriately. Medical claims indicating infusion administration are directly associated with therapy use. However, a physician might not bill for partial vials of medication (e.g., a 75 kg patient receiving 225 mg of infliximab would require three 100 mg vials) and our operational definition of infusion dose, which is based on the number of units billed for, is likely to provide an overestimation of the number of units administered. Patients in this study were not randomly assigned to therapy and factors affecting the selection of therapy or the choice to receive subsequent-line biologic therapy could result in selection bias. Previous studies indicate that discontinuation or changes in treatment are often attributable to inefficacy or adverse events, and these reasons could influence the choice of, or effectiveness of, subsequent therapy [21,23,32]. Although we are able to observe changes in treatment with claims data, the reasons for adjusting or discontinuing treatment and thus their possible effects on patient outcomes are unknown. The impact of concomitant medication use on study outcomes was not assessed. Coding errors could affect our ability to detect all relevant health care utilization. Finally, our study sample represents working-aged managed care enrollees and the results may not generalize to other patients.

## Conclusions

Although the starting doses for biologic therapies for RA are in accordance with the label recommendations, a substantial proportion of patients who received infliximab had evidence of a dose escalation during follow-up and subsequent-line anti-TNF therapy cohorts had higher rates of discontinuation, dose escalation, and shorter time to discontinuation than first-line anti-TNF cohorts. The role of biologic therapies in the treatment of RA continues to evolve, and further research is needed to determine the optimal sequence for implementing treatments with varied mechanisms of action, based on patient characteristics, response and tolerance to previous agents and comparative effectiveness of various biologic agents.

## Declaration of Competing interests

Drs. Ogale and Hitraya are employees of Genentech, and Dr. Henk is an employee of OptumInsight (formerly Innovus), an independent research organization which was contracted by Genentech to conduct the study.

## Authors' contributions

SO conceived of the study and participated in its design and data interpretation. EH made substantial contributions to the data interpretation. HJH designed the study, coordinated the analyses, and interpreted the data. All authors participated in revising the manuscript for intellectual content, and have read and approved the final manuscript.

## Pre-publication history

The pre-publication history for this paper can be accessed here:

http://www.biomedcentral.com/1471-2474/12/204/prepub

## Supplementary Material

Additional file 1 **Table S1. Exclusionary diagnosis codes**. List of diagnosis codes for conditions other than rheumatoid arthritis for which the study biologics could be prescribed. Click here for file
