# Supplementary material for: Patterns of biologic agent utilization among patients with rheumatoid arthritis: a retrospective cohort study
Source: BMC Musculoskelet Disord. 2011 Sep 19;12:204. doi: 10.1186/1471-2474-12-204 (PMC3184114; doi:10.1186/1471-2474-12-204)
Supplement: Additional file 1 — Table S1. Exclusionary diagnosis codes. List of diagnosis codes for conditions other than rheumatoid arthritis for which the study biologics could be prescribed. [file 1471-2474-12-204-S1.DOC]

Additional File 1

Table S1. Exclusionary diagnosis codes

| Condition | ICD-9-CM code |
| --- | --- |
| Non-Hodgkin’s lymphoma | 200.xx, 202.xx |
| Chronic lymphocytic leukemia | 204.10 |
| Ankylosing spondylitis | 720.0 |
| Psoriasis | 696.1 |
| Psoriatic arthritis | 696.0 |
| Crohn’s disease | 555.x |
| Ulcerative colitis | 556.x |
| Multiple sclerosis | 340.xx |
| Lupus | 710.0x |

ICD-9-CM, International Classification of Disease, 9th edition, Clinical Modification.
